# Supplementary material for: Population genomics reveals possible genetic evidence for parallel evolution of Sebastiscus marmoratus in the northwestern Pacific Ocean
Source: Open Biol. 2019 Sep 4;9(9):190028. doi: 10.1098/rsob.190028 (PMC6769290; doi:10.1098/rsob.190028)
Supplement: Supplementary Table S1; Supplementary Table S2; Supplementary Table S3; Supplementary Note [file rsob190028supp1.pdf]

Supplementary information

Population genomics reveals possible genetic evidence for parallel evolution of *Sebastiscus marmoratus* in northwestern Pacific

Shengyong Xu, Takashi Yanagimoto, Na Song, Shanshan Cai, Tianxiang Gao, Xiumei Zhang

Contents:

Supplementary Table S1

Supplementary Table S2

Supplementary Table S3

Supplementary Note

Table S1 Statistics describing different properties of each sequenced individual								
ID	Pop	Raw bases	Clean bases	Error rate	Q20	Q30	GC content	Enzyme catch ratio
F1	FCG	279,297,216	279,297,216	0.03	96.50	91.45	40.81	98.8
F2	FCG	512,185,536	512,185,536	0.03	96.74	92.03	40.63	98.8
F3	FCG	536,178,528	536,178,240	0.03	96.64	91.75	40.99	98.5
F5	FCG	488,135,808	488,135,520	0.03	96.09	90.46	41.28	98.6
F6	FCG	464,407,200	464,406,912	0.03	96.78	92.13	41.45	98.7
F7	FCG	584,929,152	584,929,152	0.03	96.51	91.52	41.08	98.5
F8	FCG	602,234,496	602,234,496	0.03	96.72	91.95	41.48	98.7
F9	FCG	596,807,136	596,806,560	0.03	96.70	91.92	41.30	98.1
F10	FCG	509,468,256	509,467,968	0.03	96.37	91.14	40.48	98.5
F11	FCG	390,631,680	390,631,680	0.03	96.12	90.65	40.83	98.3
F12	FCG	626,775,840	626,775,840	0.03	96.41	91.14	41.12	98.1
F13	FCG	547,136,352	547,135,488	0.03	96.33	91.00	41.30	98.6
F15	FCG	565,199,712	565,199,424	0.03	96.75	92.09	41.37	98.8
F16	FCG	621,485,568	621,485,568	0.03	96.65	91.81	41.31	98.8
F17	FCG	638,371,296	638,371,296	0.03	96.60	91.71	41.23	98.8
F18	FCG	628,187,616	628,187,616	0.03	96.72	92.03	41.49	98.7
F19	FCG	644,270,688	644,270,688	0.03	96.72	91.98	41.45	98.8
F21	FCG	652,987,296	652,987,296	0.03	96.13	90.47	41.44	98.7
F22	FCG	570,334,176	570,334,176	0.03	96.34	91.03	41.28	98.3
F29	FCG	514,808,640	514,794,240	0.02	97.12	93.16	41.12	99.2
R1	RS	613,854,432	613,852,992	0.01	97.91	95.15	41.36	98.6
R2	RS	372,028,896	372,016,512	0.02	97.28	93.46	41.21	99.4
R6	RS	325,704,384	325,694,016	0.02	97.28	93.42	41.41	99.4
R8	RS	331,696,512	331,687,584	0.02	96.90	92.53	41.33	99.4
R9	RS	366,242,976	366,230,592	0.01	97.42	93.81	41.57	99.5
R10	RS	341,650,080	341,638,848	0.02	97.25	93.38	41.50	99.3
R11	RS	369,481,248	369,469,728	0.02	97.32	93.56	41.50	99.4
R12	RS	295,439,904	295,433,280	0.02	97.39	93.69	41.50	99.1
R13	RS	361,205,568	361,194,912	0.02	97.07	92.97	40.77	99.3
R14	RS	333,597,024	333,586,368	0.02	96.75	92.37	40.43	99.2
R15	RS	428,501,376	428,491,872	0.02	96.93	92.62	41.28	99.2
R16	RS	353,727,360	353,712,960	0.02	97.41	93.73	41.37	99.5
R17	RS	404,602,848	404,594,784	0.02	96.91	92.63	41.42	99.4
R18	RS	465,883,776	465,871,392	0.02	97.12	93.08	41.71	99.5
R20	RS	461,024,064	461,008,224	0.01	97.25	93.46	41.86	99.5
R22	RS	367,039,872	367,031,232	0.02	97.25	93.42	41.51	99.5
R23	RS	420,722,784	420,710,400	0.02	97.10	93.09	41.43	99.5
R26	RS	433,393,344	433,381,248	0.01	97.25	93.50	41.89	99.5
R29	RS	460,085,184	460,071,360	0.02	97.28	93.48	41.78	99.5
R30	RS	419,628,384	419,620,032	0.02	96.65	91.98	41.28	99.4
Z1	ZS	723,848,832	723,846,528	0.01	97.72	94.84	40.44	98.5

Z2	ZS	662,437,728	662,436,864	0.01	97.94	95.23	41.09	98.6
Z3	ZS	597,074,976	597,074,400	0.01	97.90	95.15	41.03	98.5
Z4	ZS	668,546,784	668,545,920	0.01	97.91	95.23	40.95	98.5
Z7	ZS	657,551,232	657,549,792	0.01	97.89	95.22	40.69	98.7
Z8	ZS	624,568,320	624,567,168	0.01	97.93	95.24	41.13	98.4
Z9	ZS	610,747,776	610,747,488	0.01	97.68	94.73	40.30	98.2
Z10	ZS	655,331,616	655,330,464	0.01	97.94	95.18	41.02	98
Z11	ZS	591,719,328	591,719,040	0.01	97.78	94.81	41.13	98.5
Z12	ZS	612,705,888	612,704,448	0.01	97.81	95.11	40.75	97.8
Z13	ZS	643,852,224	643,851,648	0.01	97.69	94.84	40.48	98.3
Z14	ZS	427,900,320	427,900,320	0.01	97.67	94.81	40.14	98.3
Z18	ZS	684,231,840	684,229,536	0.01	97.79	95.03	40.42	97.1
Z20	ZS	581,005,152	581,004,288	0.01	97.79	94.97	40.52	98.3
Z31	ZS	490,484,448	490,484,448	0.03	96.39	91.26	40.48	98.7
Z32	ZS	742,958,784	742,958,784	0.03	96.56	91.59	41.17	98.7
Z34	ZS	1,154,939,040	1,154,884,320	0.02	97.89	94.78	41.07	99.4
Z35	ZS	604,194,048	604,194,048	0.03	96.42	91.25	41.06	98.7
Z36	ZS	563,654,304	563,654,016	0.03	96.67	91.86	40.98	98.8
Z37	ZS	374,124,960	374,112,864	0.02	97.12	93.07	41.41	99.2
J1	NI	231,678,144	231,664,896	0.05	93.87	85.00	39.55	90.6
J2	NI	270,722,016	270,703,296	0.05	94.02	85.00	41.19	90.4
J3	NI	260,223,552	260,209,152	0.06	93.94	85.00	40.66	90.5
J4	NI	279,980,928	279,961,920	0.05	93.79	85.00	41.64	90.6
J5	NI	286,279,200	286,260,192	0.04	94.40	86.52	40.66	91.7
J6	NI	287,759,520	287,744,544	0.04	94.05	85.67	40.27	91.1
J7	NI	343,851,264	343,827,936	0.04	94.09	85.75	39.05	91.1
J8	NI	318,367,296	318,344,256	0.04	94.44	86.62	40.48	91.7
J9	NI	354,643,488	354,622,752	0.04	94.43	86.44	39.99	91.5
J10	NI	268,125,696	268,110,432	0.06	94.09	85.00	40.28	90.3
X1	XM	250,911,072	250,897,824	0.06	93.94	85.00	41.41	90.5
X10	XM	253,240,704	253,225,728	0.05	93.86	85.25	40.66	90.8
X2	XM	333,902,304	333,884,448	0.04	94.25	86.00	40.83	91.2
X3	XM	338,505,408	338,491,008	0.05	93.97	85.00	39.52	90.5
X4	XM	266,237,280	266,218,272	0.04	94.41	86.56	41.03	91.7
X5	XM	285,449,184	285,428,736	0.05	94.32	86.40	40.77	91.6
X6	XM	294,747,552	294,729,408	0.04	94.09	85.70	40.50	91.1
X7	XM	301,586,400	301,570,272	0.04	94.22	86.05	40.57	91.3
X8	XM	265,672,512	265,659,840	0.06	94.00	85.00	40.92	90.4
X9	XM	254,359,584	254,347,776	0.05	94.11	85.00	39.45	90.3
G12	KO	438,953,184	438,952,608	0.03	96.53	91.24	41.7	99.3
G13	KO	550,803,744	550,803,168	0.03	96.27	90.6	41.01	99.2
G14	KO	475,020,864	475,018,848	0.03	96.65	91.55	41.39	99.4
G15	KO	574,030,368	574,029,792	0.03	96.01	90.06	40.59	99.3

G16	KO	473,412,960	473,412,672	0.03	96.69	91.82	41.55	99.4
G18	KO	387,787,968	387,786,240	0.03	96.68	91.78	40.27	99.4
G20	KO	429,270,048	429,268,608	0.03	96.55	91.32	41.41	99.3
G21	KO	411,142,176	411,141,312	0.03	96.5	91.39	40.6	99.2
G22	KO	162,223,200	162,222,624	0.03	95.95	90.08	39.47	99.1
G4	KO	211,487,040	211,486,752	0.03	96.47	91.34	40.68	99.2
Y11	YO	454,484,448	454,483,584	0.03	96.27	90.72	40.98	99.2
Y12	YO	225,791,712	225,791,424	0.03	96.38	91.11	40.76	99.2
Y13	YO	471,962,880	471,961,152	0.03	96.67	91.73	41.34	99.4
Y15	YO	525,339,072	525,337,056	0.03	96.51	91.28	41.45	99.3
Y17	YO	322,540,992	322,539,264	0.03	96.29	90.94	40.59	99.4
Y18	YO	290,228,256	290,227,680	0.03	96.47	91.41	40.77	99.4
Y19	YO	550,646,208	550,645,056	0.03	96.68	91.66	41.44	99.1
Y22	YO	209,812,032	209,812,032	0.03	95.66	89.34	39.33	99.1
Y3	YO	174,844,800	174,844,512	0.03	96.13	90.56	40.56	99.1
Y9	YO	276,379,200	276,378,912	0.03	96.34	90.98	40.86	99.2
N1	TO	462,798,432	462,798,144	0.03	95.92	89.77	41.5	99.4
N10	TO	474,031,296	474,030,144	0.03	96.16	90.38	41.7	99.4
N11	TO	470,651,040	470,650,752	0.03	96.47	91.09	42.08	99.5
N3	TO	411,908,256	411,907,392	0.03	96.66	91.74	41.2	99.5
N4	TO	433,440,288	433,439,136	0.03	96.33	90.89	41.49	99.3
N5	TO	429,002,208	429,000,768	0.03	96.61	91.48	41.62	99.5
N6	TO	501,343,488	501,341,760	0.03	96.58	91.42	41.53	99.2
N7	TO	353,541,600	353,540,736	0.03	96.16	90.41	41.02	99.4
N8	TO	367,263,072	367,262,496	0.03	96.05	90.22	40.75	99.2
N9	TO	479,788,992	479,788,704	0.03	96.24	90.48	41.6	99.3
Y1	IK	588,763,008	588,662,784	0.02	96.96	92.15	42.06	99
Y10	IK	616,333,248	616,240,512	0.03	96.15	90.23	41.41	98.6
Y13	IK	528,535,008	528,457,536	0.03	96.58	91.22	41.1	98.6
Y14	IK	466,936,704	466,856,640	0.03	96.83	91.87	41.48	98.9
Y15	IK	706,539,168	706,415,616	0.03	96.69	91.58	41.75	98.8
Y20	IK	398,496,960	398,427,264	0.03	96.73	91.91	41.2	98.7
Y22	IK	524,778,336	524,693,376	0.03	96.56	91.48	41.11	98.8
Y3	IK	537,937,344	537,844,608	0.03	96.6	91.52	41.05	98.8
Y4	IK	679,418,208	679,305,024	0.03	96.59	91.66	41.14	98.9
Y6	IK	579,987,360	579,898,080	0.03	96.77	91.89	41.59	98.9
H13	ZH	788,605,920	788,471,424	0.03	97.02	92.6	41.41	99
H14	ZH	672,548,832	672,435,072	0.03	96.69	91.84	40.56	98.7
H17	ZH	699,660,288	699,554,880	0.03	96.94	92.23	41.39	98.6
H18	ZH	428,307,264	428,237,280	0.02	96.95	92.48	40.52	98.5
H21	ZH	629,594,496	629,496,288	0.03	96.5	91.05	41.61	98.8
H22	ZH	668,142,144	668,039,904	0.03	96.36	90.74	41.3	98.9

H24	ZH	653,014,656	652,916,160	0.03	96.56	91.36	40.89	98.7
H3	ZH	465,552,864	465,480,864	0.03	96.53	91.19	41.76	98.6
H5	ZH	565,880,256	565,785,216	0.03	96.83	92	40.88	98.7
H9	ZH	777,025,152	776,900,736	0.03	96.55	91.16	41.22	98.9

Supplementary Table S2 Descriptions of blast results and gene annotation

	Description	Length	Hits	e-Value	sim mean	GO Names list
Contig 158238	polypeptide N-acetylgalactosaminyltransferase 18 isoform X2	210	20	4.34E-34	99.80%	F:transferase activity, transferring glycosyl groups; C:integral component of membrane; P:protein glycosylation; C:Golgi membrane; F:carbohydrate binding
Contig 368320	dnaJ homolog subfamily B member 11	308	20	8.00E-11	95.35%	F:unfolded protein binding; P:protein folding
Contig 43470	39S ribosomal mitochondrial	174	20	1.62E-16	94.95%	F:structural constituent of ribosome; C:mitochondrion; P:ribosome biogenesis; C:ribosome; P:translation
Contig 545705	myosin heavy fast skeletal muscle-	145	20	1.01E-08	94.60%	C:myosin complex; F:motor activity
Contig 522430	disintegrin and metallo ase domain-containing 10-like	308	20	1.53E-18	89.00%	F:zinc ion binding; P:integrin-mediated signaling pathway; P:Notch signaling pathway; P:membrane protein ectodomain proteolysis; C:integral component of membrane; F:metalloendopeptidase activity
Contig 321865	RNA-directed DNA polymerase from mobile element jockey-	202	20	4.37E-20	88.65%	F:RNA-directed DNA polymerase activity; C:voltage-gated calcium channel complex; P:calcium ion transmembrane transport; P:RNA-dependent DNA biosynthetic process; F:voltage-gated calcium channel activity
Contig 160553	crumbs homolog 2-like	195	20	4.96E-17	75.25%	F:oxidoreductase activity, acting on paired donors, with incorporation or reduction of molecular oxygen, 2-oxoglutarate as one donor, and incorporation of one atom each of oxygen into both donors; C:integral component of membrane; F:methyltransferase activity; P:oxidation-reduction process; P:methylation
Contig 264384	LINE-1 reverse transcriptase	257	15	3.25E-09	73.27%	P:cortical actin cytoskeleton organization; F:RNA-directed DNA polymerase activity; C:cytoskeleton; F:actin binding; F:structural molecule activity; P:RNA-dependent DNA biosynthetic process; F:cytoskeletal protein binding

Supplementary Table S3 Genetic diversity estimates of subsampled populations using neutral loci

Site code	Nucleotide diversity	Observed heterozygosity	Expected heterozygosity
NI	0.112±0.056	0.174±0.128	0.170±0.097
KO	0.082±0.041	0.172±0.147	0.165±0.105
YO	0.075±0.038	0.176±0.159	0.166±0.108
TO	0.074±0.037	0.171±0.149	0.162±0.106
IK	0.116±0.058	0.179±0.129	0.175±0.099
RS	0.179±0.089	0.201±0.121	0.217±0.107
ZS	0.225±0.112	0.229±0.127	0.246±0.111
XM	0.115±0.057	0.175±0.123	0.172±0.098
ZH	0.124±0.062	0.180±0.123	0.178±0.099
FCG	0.141±0.070	0.185±0.117	0.190±0.104

Supplementary Note

The parameter scripts of BWA, SAMtools and VCFtools in the present study were as following:

BWA: bwa *index* –a is; bwa *mem*;

SAMtools: samtools *view* –S –b; *sort* –l 0; *mpileup* -B -C 50 -g -u -D -q 0 -Q 13 –I | bcftools view -bvcg -;

VCFtools: vcfutils.pl varFilter -Q 30 -d 10 -D 200; vcftools --recode --out --maf 0.05 --hwe 0.05 --min-alleles 2 --max-alleles 2 --max-missing 0.9 --remove-indels.
